# Comprehensive Physicochemical Characterization, In Vitro Membrane Permeation, and In Vitro Human Skin Cell Culture of a Novel TOPK Inhibitor, HI-TOPK-032

**DOI:** 10.3390/ijms242115515

**Published:** 2023-10-24

**Authors:** Basanth Babu Eedara, Bhagyashree Manivannan, Wafaa Alabsi, Bo Sun, Clara Curiel-Lewandrowski, Tianshun Zhang, Ann M. Bode, Heidi M. Mansour

**Affiliations:** 1 Center for Translational Science, Florida International University, Port St. Lucie, FL 34987, USA; babubasanth@gmail.com (B.B.E.); bmanivan@fiu.edu (B.M.); 2 Skaggs Pharmaceutical Sciences Center, College of Pharmacy, The University of Arizona, Tucson, AZ 85721, USA; wafaaalabsi@arizona.edu (W.A.); bsun168@arizona.edu (B.S.); 3 Department of Chemistry and Biochemistry, The University of Arizona, Tucson, AZ 85721, USA; 4 Skin Cancer Institute, The University of Arizona Cancer Center, Tucson, AZ 85724, USA; ccuriel@arizona.edu; 5 University of Arizona Cancer Center, University of Arizona, Tucson, AZ 85724, USA; 6 Department of Medicine, Division of Dermatology, College of Medicine, The University of Arizona, Tucson, AZ 85724, USA; 7 The Hormel Institute, University of Minnesota, Austin, MN 55912, USA; zhan4145@umn.edu (T.Z.); bodex008@umn.edu (A.M.B.); 8 Department of Environmental Health Sciences, Robert Stempel College of Public Health and Social Work, Florida International University, Miami, FL 33199, USA; 9 Department of Cell Biology & Pharmacology, Herbert Wertheim College of Medicine, Florida International University, Miami, FL 33199, USA

**Keywords:** NMSC, Strat-M, HaCaT cell line, NHEK cells, cell viability, TEER, air–liquid interface

## Abstract

Nonmelanoma skin cancers (NMSC) are the most common skin cancers, and about 5.4 million people are diagnosed each year in the United States. A newly developed T-lymphokine-activated killer cell-originated protein kinase (TOPK) inhibitor, HI-TOPK-032, is effective in suppressing colon cancer cell growth, inducing the apoptosis of colon cancer cells and ultraviolet (UV) light-induced squamous cell carcinoma (SCC). This study aimed to investigate the physicochemical properties, permeation behavior, and cytotoxicity potential of HI-TOPK-032 prior to the development of a suitable topical formulation for targeted skin drug delivery. Techniques such as scanning electron microscopy (SEM), energy-dispersive X-ray (EDX) spectroscopy, differential scanning calorimetry (DSC), hot-stage microscopy (HSM), X-ray powder diffraction (XRPD), Karl Fisher (KF) coulometric titration, Raman spectrometry, confocal Raman microscopy (CRM), attenuated total reflectance-Fourier transform infrared spectroscopy (ATR-FTIR), and Fourier transform infrared microscopy were used to characterize HI-TOPK-032. The dose effect of HI-TOPK-032 on in vitro cell viability was evaluated using a 2D cell culture of the human skin keratinocyte cell line (HaCaT) and primary normal human epidermal keratinocytes (NHEKs). Transepithelial electrical resistance (TEER) at the air–liquid interface as a function of dose and time was measured on the HaCAT human skin cell line. The membrane permeation behavior of HI-TOPK-032 was tested using the Strat-M^®^ synthetic biomimetic membrane with an in vitro Franz cell diffusion system. The physicochemical evaluation results confirmed the amorphous nature of the drug and the homogeneity of the sample with all characteristic chemical peaks. The in vitro cell viability assay results confirmed 100% cell viability up to 10 µM of HI-TOPK-032. Further, a rapid, specific, precise, and validated reverse phase-high performance liquid chromatography (RP-HPLC) method for the quantitative estimation of HI-TOPK-032 was developed. This is the first systematic and comprehensive characterization of HI-TOPK-032 and a report of these findings.

## 1. Introduction

Skin cancer is the most common cancer in the United States (US) [1]. The American Academy of Dermatology estimates the diagnosis of about 9500 skin cancer cases in US every day [2]. Nonmelanoma skin cancers (NMSC), including basal cell carcinoma (BCC) and squamous cell carcinoma (SCC), are the most common skin cancers and are estimated to affect about 5.4 million people every year in the US [3]. Exposure to ultraviolet (UV) radiation from the sun is a major environmental carcinogen for approximately 98% of skin cancers.

TOPK (T-lymphokine-activated killer cell-originated protein kinase), a member of the mitogen-activated protein kinase (MAPK) protein family [4], is involved in many cellular functions, including tumor development, cell growth, apoptosis, and inflammation [5,6]. TOPK is highly expressed in many human cancers, such as leukemia, lymphoma, myeloma, breast cancer, and colorectal cancer [6,7,8,9,10,11]. Roh et al. [12] reported that acute UV irradiation increases the protein and phosphorylation levels of TOPK in human skin tissue. Thus, TOPK could be a promising molecular target for the prevention and control of UV-induced skin cancer [6].

**Figure 1:**
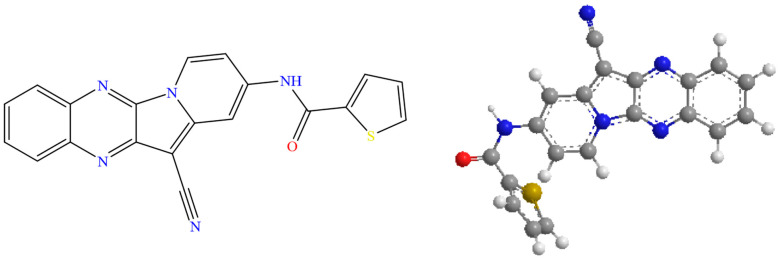
Chemical structures (drawn using Chem Draw^®^ Ver. 21.0.0, CambridgeSoft, Cambridge, MA, USA) of HI-TOPK-032.

At present, three TOPK-targeted and specific inhibitors have been developed as follows: HI-TOPK-032, OTS514/OTS964, and ADA-07 [13]. HI-TOPK-032 (N-(12-cyanoindolizino[2,3-b] quinoxalin-2-yl)-2-thiophenecarboxamide. Figure 1) directly inhibits TOPK activity in vitro and in vivo, and is effective in suppressing colon cancer cell growth and inducing the apoptosis of colon cancer cells [14]. Recently, Roh et al. [15] demonstrated that HI-TOPK-032 can suppress UV-induced SCC through the TOPK-c–Jun axis and its topical application can be used as a potential chemopreventive drug against SCC development.

Prior to the development of various topical formulations of HI-TOPK-032, the comprehensive characterization of raw drugs to understand the physicochemical nature of the drug, permeation behavior, and cytotoxicity of this drug to human keratinocyte skin cells is necessary. The comprehensive physicochemical characterization of raw HI-TOPK-032 includes residual water content estimation using Karl Fisher coulometric titration (KFT), particle size and surface morphology using scanning electron microscopy (SEM), a solid-state nature using X-ray powder diffraction (XRPD) analysis, thermal behavior using differential scanning calorimetry, hot-stage microscopy, and molecular fingerprinting using Raman spectroscopy and attenuated total reflectance-Fourier transform infrared spectroscopy. The cytotoxicity potential of HI-TOPK-032 with increasing drug concentration (0.1 µM to 1000 µM) was evaluated using a 2D cell culture of the HaCaT human keratinocyte skin cell line. The permeation behavior of HI-TOPK-032 was determined using Strat-M^®^ synthetic biomimetic membrane with a Franz cell diffusion system. Furthermore, a sensitive, reproducible, and reliable analytical method was required for the estimation of HI-TOPK-032 concentration. To the best of our knowledge, no chromatographic methods have been reported for the quantification of HI-TOPK-032. In this study, a rapid, specific, precise, and validated reverse phase-high performance liquid chromatographic (RP-HPLC) method for the quantitative estimation of HI-TOPK-032 is reported.

## 2. Results

### 2.1. Physicochemical Characterization

#### 2.1.1. Scanning Electron Microscopy (SEM) and Energy-Dispersive X-ray (EDX) Spectroscopy

**Figure 2:**
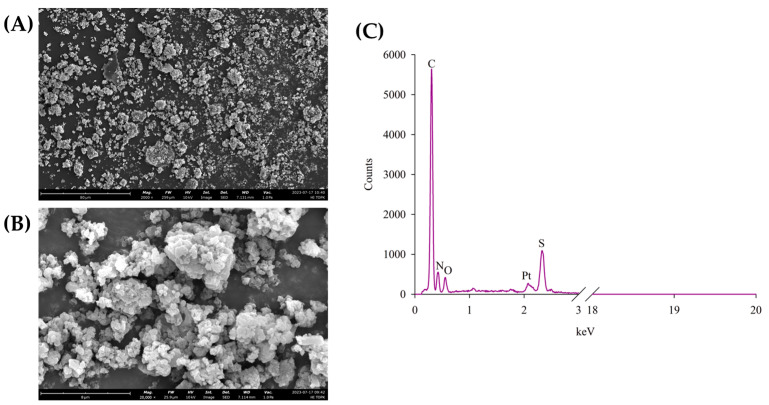
Representative scanning electron microscopic (SEM) images of raw HI-TOPK-032 at 2000× (**A**) and 20,000× (**B**) magnifications, and the energy-dispersive X-ray (EDX) spectrum (**C**) of raw HI-TOPK-032 showing characteristic elemental peaks.

The SEM micrographs of HI-TOPK-032 (Figure 2A,B) showed irregular-shaped, aggregated particles with varying sizes and rough surfaces. The EDX spectrum (Figure 2C) of HI-TOPK-032 showed carbon (C), nitrogen (N), oxygen (O), and sulfur (S) elements along with platinum from the coating.

#### 2.1.2. Differential Scanning Calorimetry (DSC)

**Figure 3:**
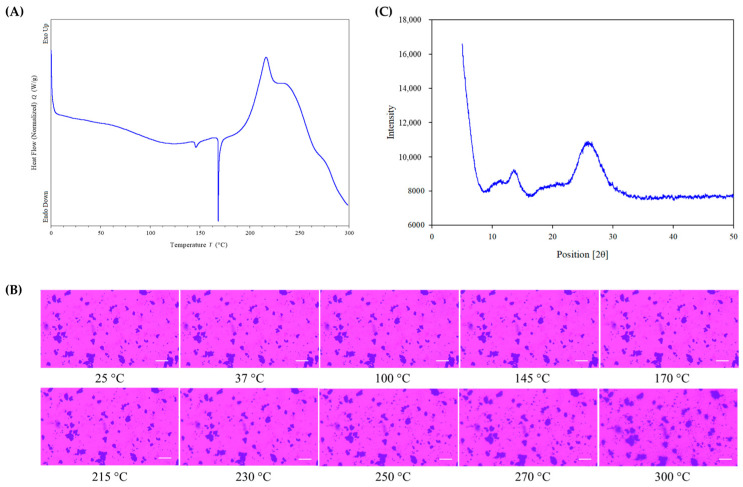
(**A**) Differential scanning calorimetry (DSC) thermogram of raw HI-TOPK-032 (*n* = 4); (**B**) Representative hot stage microscopy (HSM) images of raw HI-TOPK-032 (Scale bar = 50 µm, *n* = 3); (**C**) X-ray powder diffractograms (XRPD) of raw HI-TOPK-032 (*n* = 3).

The raw HI-TOPK-032 DSC thermogram (Figure 3A) exhibited two endothermic peaks at ~145 °C and ~171 °C and a third exothermic peak at ~216 °C (Table 1). These observed peaks are a unique feature of raw HI-TOPK-032 red amorphous powder.

#### 2.1.3. Hot-Stage Microscopy (HSM)

As visualized by cross-polarized light microscopy, raw HI-TOPK-032 (Figure 3B) exhibited an absence of birefringence, indicating the amorphous nature of the drug. No visual changes or melting of the drug were observed upon heating up to 300 °C.

#### 2.1.4. X-ray Powder Diffraction (XRPD)

The XRPD diffractogram of HI-TOPK-032 (Figure 3C) exhibited a typical halo with an absence of diffraction peaks, indicating the lack of long-range molecular order and the amorphous nature of the drug.

#### 2.1.5. Karl Fisher (KF) Coulometric Titration

The residual water content of the raw HI-TOPK-032 was found to be 2.464 ± 0.235% (*w*/*w*) (*n* = 3, mean ± standard deviation). The low water content of the raw HI-TOPK-032 was consistent with the hydrophobic nature of the drug.

#### 2.1.6. Raman Spectrometry and Confocal Raman Microscopy (CRM)

**Figure 4:**
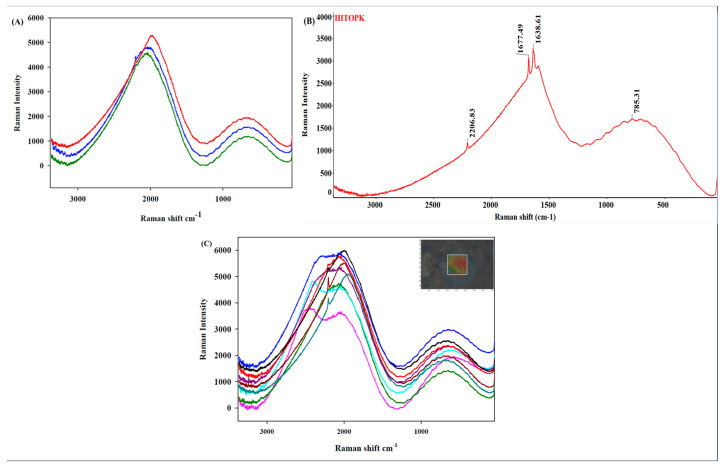
(**A**) Raman spectra (*n* = 3) of raw HI-TOPK-032 using 785 nm laser; (**B**) Representative HI-TOPK-032 Raman spectrum with peak values (*n* = 3); (**C**) Raman microscopy mapping of raw HI-TOPK-032 showing 9 spectra using 785 nm laser and Raman map (inset) with the area (white box) measured via the spectroscopic mapping method (*n* = 3).

Characteristic peaks were identified using Raman spectrometry (Figure 4A) for raw HI-TOPK-032 and showed four prominent peaks (Figure 4B) representing 785.31 cm^−1^ (C-C functional group), 1638.61 cm^−1^ and 1677.49 cm^−1^ (>C=O mixed with NH deformation) and 2206.83 cm^−1^ (C≡C functional group). Raman microscopy mapping demonstrated the homogeneity of the sample (Figure 4C).

#### 2.1.7. Attenuated Total Reflectance-Fourier Transform Infrared Spectroscopy (ATR-FTIR) and Fourier Transform Infrared Microscopy

**Figure 5:**
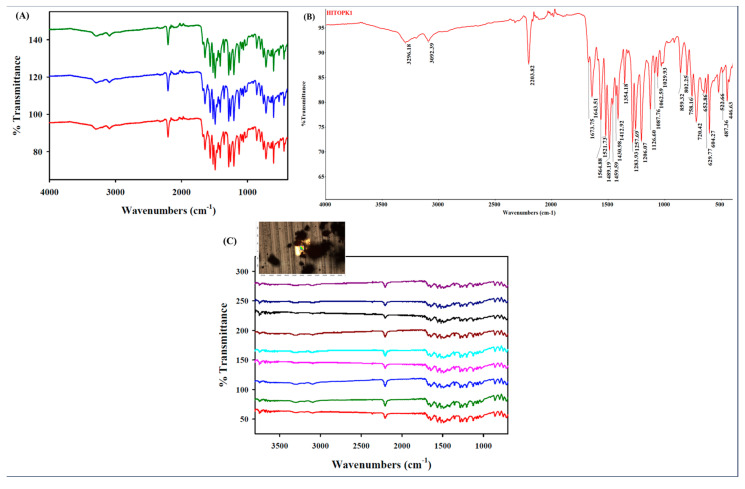
(**A**) ATR-FTIR spectrum of raw HI-TOPK-032 (*n* = 3; samples 1–3 represent spectra from three individual samples); (**B**) Representative HI-TOPK-032 FTIR spectrum with peak values (*n* = 3). (**C**) FTIR microscopy mapping of raw HI-TOPK-032 showing 9 spectra and an FTIR map (inset) with the approximate area (red box) measured by the spectroscopic mapping method (*n* = 3).

The ATR-FTIR spectrum (Figure 5A,B) of raw HI-TOPK-032 showed a prominent peak at 2203.82 cm^−1^ (C≡N functional group) with a consistent spectral pattern seen at the fingerprint region (<2000 cm^−1^). The IR microscopy chemical map (Figure 5C) was consistent with the results obtained using FTIR spectrometry. Table 2 shows the important characteristic chemical peaks of HI-TOPK-032, which were identified via Raman spectrometry and FTIR spectrometry.

### 2.2. In Vitro 2D Cell Culture Dose–Response Assay with HaCaT and NHEK Cells

**Figure 6:**
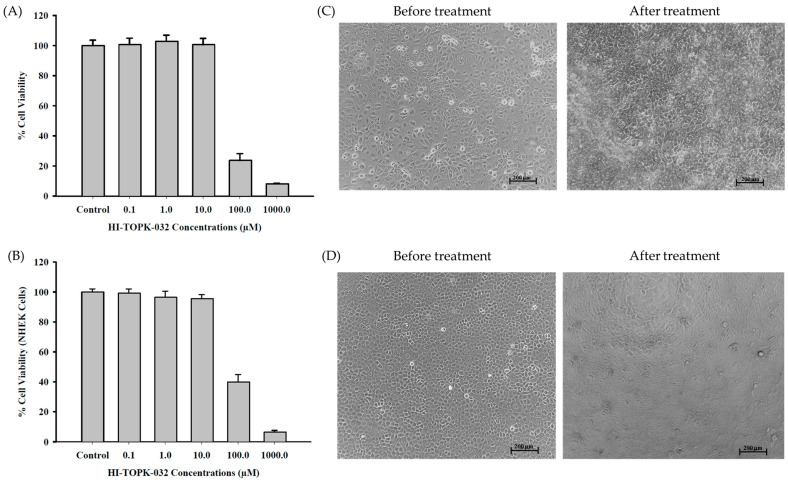
In vitro cell viability assay using (**A**) HaCaT cells (immortalized transformed human keratinocyte cell line) and (**B**) NHEK cells (normal human epidermal keratinocytes) and dose response with different concentrations of raw HI-TOPK-032 (*n* = 24 for each concentration). Representative images of the microscopic examination of HaCat (**C**) and NHEK (**D**) cell morphology before and after treatment with HI-TOPK-032.

In vitro cell viability assays were conducted using HaCaT (Figure 6A) and NHEK cells (Figure 6B) with increasing HI-TOPK-032 concentrations and an exposure time of 48 h showed 100% viability at 0 µM (control), 0.1 µM, 1 µM, and 10 µM, and decreased viability with 100 µM (HaCaT- ≈27%; NHEK- ~39%) and 1000 µM (HaCaT- ≈8%; NHEK- ~6%) of the drug concentration, respectively.

HaCat cells were cultured (Figure 6C) in supplemented ADMEM (before treatment) and non-supplemented ADMEM (during HI-TOPK-032 treatments) with a simple microscopic observation showing a cuboidal and stratified shape with close packing from monolayer to multilayer at 48 h of exposure to the drug. Further visual inspection confirmed cell rounding, cytoplasmic vacuolation, and cell debris particles at concentrations of 100 µM and 1000 µM.

NHEK cells (Figure 6D) maintained in supplemented KGM (before treatment) and non-supplemented KGM (during HI-TOPK-032 treatments) upon microscopic observation showed a typical cobblestone-like morphology with proliferation to a multilayer morphology at 48 h of exposure time. Concentrations of 100 µM and 1000 µM showed observable cell rounding and floating cell debris using light microscopy.

### 2.3. In Vitro Transepithelial Electrical Resistance (TEER)

**Figure 7:**
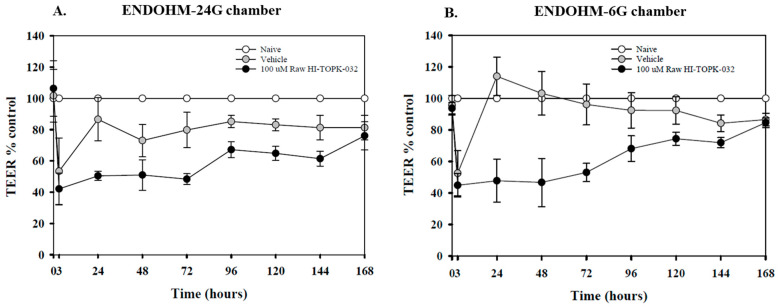
In vitro TEER values recorded using ENDOHM-24G (**A**) and ENDOHM-6G (**B**) chambers, of HaCaT cells at air–liquid interface (ALI) conditions exposed to 100 µM of a raw HI-TOPK-032 concentration for 3 h. TEER values were recorded before and after 3 h of HI-TOPK-032 exposure and subsequently at 24 h until 7 days. Data shown are the TEER values calculated as the percentage response of the control/naïve (non-treated) values using *n* = 4 replicates.

The in vitro TEER values recorded over a period of 7 days for the HaCaT immortalized and transformed keratinocyte cells, either naïve and treated with 100 µM of a raw HI-TOPK-032 concentration for 3 h, using ENDOHM-24G (Figure 7A) and ENOHM-6G (Figure 7B) chambers, indicating that treated cells (either HI-TOPK-032 or DMSO treated) recovered gradually over a period when compared to naïve cells.

### 2.4. In Vitro Permeation of HI-TOPK-032 through Strat-M^®^ Transdermal Diffusion Membrane

**Figure 8:**
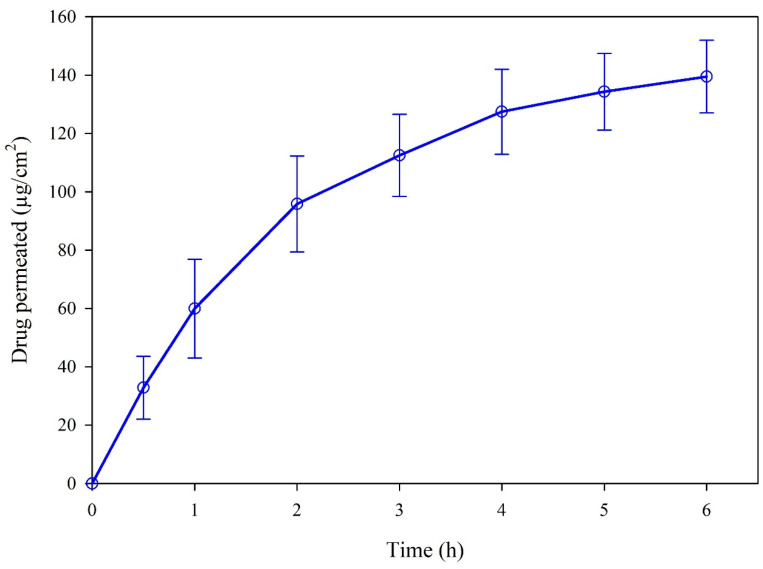
In vitro Franz-cell/Strat-M^®^ permeation profile of HI-TOPK-032 (*n* = 12, mean ± standard deviation).

The in vitro permeation behavior of HI-TOPK-032 from a propylene glycol solution through the Strat-M transdermal diffusion membrane was evaluated using a Franz diffusion system. Figure 8 shows the increased permeation of HI-TOPK-032 over 6 h using the Strat-M membrane without any lag phase. At the end of 6 h, the permeation of HI-TOPK-032 was found to be 139.5 ± 12.4 µg/cm^2^ with a steady state flux of 0.0241 ± 0.0023 µg/cm^2^/h.

### 2.5. HPLC Method Development, Optimization, and Validation

**Figure 9:**
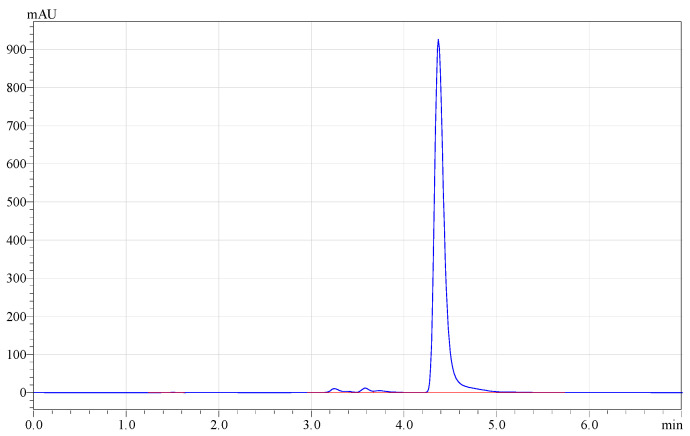
RP-HPLC chromatogram of HI-TOPK-032 (retention time = 4.3733 min) in a standard drug solution of 5 µg/mL.

Hi-TOPK-032 is hydrophobic and is almost insoluble in water and ethanol. It has a solubility of 4 mg/mL in DMSO. Thus, a primary stock solution of HI-TOPK-032 was prepared using DMSO. During analytical method development, the use of methanol or acetonitrile either alone with water or as a mobile phase resulted in an asymmetric peak with a >2 tailing factor. A combination of methanol and acetonitrile at 50:50 *v*/*v* with water produced a symmetric peak with a tailing factor of 1.3362. HI-TOPK-032 was eluted at a retention time of 4.373 min with a good peak shape and symmetry at a maximum wavelength of 205 nm, as depicted in Figure 9.

#### 2.5.1. System Suitability, Linearity and Sensitivity

The system’s suitability parameters, such as peak retention time, area, height, the number of theoretical plates, and tailing factor, were determined by injecting six replicate injections of a standard HI-TOPK-032 solution of 5 µg/mL. The % CV of all the parameters was found to be within the acceptable limit of <2%, as shown in Table 3A. The developed analytical method was found to fulfill the requirements of system suitability.

The standard calibration curves for HI-TOPK-032 were found to be linear in the concentration range of 0.5 to 8.0 µg/mL with a correlation coefficient (R^2^) greater than 0.999 (Table 3B). Standard deviations of the slope and intercept for the calibration curves (*n* = 6) were 7756 and 12,582, respectively. The LOD and LOQ values were found to be 0.010 µg/mL and 0.030 µg/mL (Table 3B), respectively, indicating the high sensitivity of the developed analytical method.

#### 2.5.2. Accuracy, Precision, and Recovery

The accuracy and precision values were calculated for the QC samples during intra- and inter-day runs, as shown in Table 4. The overall % recovery for the LQC, MQC, and HQC samples at intra- and inter-day runs was found in the range of 95–103%. The % of RSD and % of bias ranged between 0.05 and 2.90% and 1.02–4.88%, respectively, which were well within the acceptance criteria of <15%. These results indicate that the developed method represents the reliable analysis of HI-TOPK-032 in quality control laboratories.

#### 2.5.3. Robustness, Carry-Over and Stability

To determine the robustness of the developed analytical method, the effect of the intended change in the mobile phase flow rate and oven temperature on peak retention time, peak area, the number of theoretical plates, tailing factor, and identified drug concentration were studied for the LQC, MQC, and HQC samples (Table 5). The slight variation (±5%) in the mobile phase flow rate and oven temperature showed a slight change in peak retention times (0.2 min with flow rate change and 0.01 min with oven temperature change) as expected. With the slight change in oven temperature and flow rate change, the tailing factor remained within acceptable limits (<2). The percentage of RSD values was found to be <2%. All the concentrations were identified with slight variation in the mobile phase flow rate, and over temperature were within the acceptable limits with a % bias <15%, indicating the robustness of the developed method.

No carry-over was found during the validation of the developed analytical method, indicating the suitability of the method for routine analysis.

The short-term stability of the drug solution under different storage conditions is shown in Table 6. The QC samples were stable when kept at bench top (~20 °C) in an auto-sampler (15 °C), refrigerator at 4 °C and freezer at −20 °C for 48 h. All the samples showed a percentage bias of <5% with the mean concentration after storage within the acceptable range of ±15% of the nominal concentration.

## 3. Discussion

HI-TOPK-032 is a newly developed TOPK inhibitor, which is effective in suppressing colon cancer cell growth and inducing the apoptosis of colon cancer cells [14. Recently, HI-TOPK-032 has been shown to suppress UV-induced SCC [15]. A detailed physicochemical characterization and solid-state analysis of a new drug molecule is necessary to understand its properties prior to the development of a suitable formulation with optimum therapeutic efficacy. This manuscript reports for the first time a comprehensive physicochemical characterization of HI-TOPK-032, such as imaging via scanning electron microscopy with energy dispersive spectroscopy, X-ray powder diffraction analysis, thermal analysis, hot-stage microscopy, the residual water content estimation using KF titration, and molecular fingerprinting via spectroscopy. The X-ray diffractogram of HI-TOPK-032 indicates the amorphous nature of the drug in its supplied form without intense diffraction peaks. Further, the amorphous nature of HI-TOPK-032 was supported by the absence of crystalline birefringence in HSM. The DSC thermogram showed two solid phase transition peaks at ~145 °C and ~171 °C and an exothermic peak at ~216 °C indicative of a disorder-to-order solid-state first-order phase transition. The minimal residual water content of HI-TOPK-032 powder, which was confirmed from KFT, is consistent with the hydrophobic nature of the drug.

The cytotoxicity potential of HI-TOPK-032 was evaluated using a 2D human cell culture of HaCaT skin cells and NHEK cells. HaCaT cells are long-lived, immortalized human keratinocytes derived from adult trunk skin and have been widely used to study epidermal homeostasis and its pathophysiology [16]. HaCaT cells are a reproducible and reliable in vitro model for studies on epidermal architecture, inflammatory/repair responses, and skin metabolism [17,18,19,20]. Further, the p53 mutations of HaCaT cells are a distinctive feature of cutaneous SCC and are used as a model for analyzing skin cancer development. Primary keratinocytes, NHEK cells, which are isolated from an adult skin epidermis, have been widely used as a model for inflammatory skin diseases and skin responses to ultraviolet radiation or oxidative stress [17,21,22,23]. The in vitro cytotoxicity potential of HI-TOPK-032 (0–1000 µM) using human skin cells (2D cell culture) was successfully demonstrated, and the results show that the viability of HaCaT and NHEK cells remained high for up to 10 µM drug dose concentrations, which indicates that the drug may be safe to use at therapeutic doses. Both HaCaT and NHEK cell viability decreased significantly at a concentration > 100 µM for the drug dose concentration, indicating a dose-dependent effect on cell viability.

Strat-M^®^ is a synthetic non-animal-based membrane model that mimics key structural and chemical features of human skin used for transdermal diffusion studies [24]. The tight top layer of the Strat-M membrane is coated with lipids simulating the lipid chemistry of the human stratum corneum (SC), and the lower porous layer simulates the epidermis and dermis layers of human skin [25]. The permeation behavior of HI-TOPK-032 from a propylene glycol solution using the Strat-M membrane was evaluated using the Franz cell diffusion system, and the results showed a linear increase without a lag phase. This demonstrates the necessity of suitable formulation development of HI-TOPK-032 for skin-targeted drug delivery with high drug retention for topical applications.

A simple, sensitive, isocratic reversed-phase HPLC method for the quantification of HI-TOPK-032 was developed and validated. This method was successfully used to evaluate the drug permeation behavior of HI-TOPK-032 through Strat-M^®^ synthetic biomimetic membrane.

## 4. Materials and Methods

### 4.1. Materials

HI-TOPK-032 (N-(12-cyanoindolizino[2,3-b] quinoxalin-2-yl)-2-thiophenecarboxamide, C_20_H_11_N_5_OS, molecular weight = 369.4 g/mol, red solid, >98% purity), as shown in Figure 1, was purchased from Bio-Techne Corporation (Minneapolis, MN, USA). Propylene glycol (PG, USP/FCC certified), HPLC-grade methanol, and acetonitrile were obtained from Fisher Scientific (Fair Lawn, NJ, USA). Dimethyl sulfoxide (DMSO, ≥99.5% (GC)), Tween^®^ 80, and Hydranal^®^-Coulomat AD were obtained from Sigma-Aldrich (St. Louis, MO, USA). The Strat-M^®^ membrane (47 mm), a synthetic, non-animal-based transdermal diffusion test model, was purchased from Millipore Sigma (Danvers, MA, USA).

Transformed keratinocytes from histologically normal human skin (HaCaT cells) were obtained from AddexBio^®^ T0020001, San Diego, CA, USA. Advanced Dulbecco’s Modified Eagle’s Medium 1′ (ADMEM, Gibco^®^), Gibco^®^ Collagen I, Rat Tail, Gibco^®^ Fetal Bovine Serum (FBS), Gibco^®^ Penicillin-Streptomycin (10,000 U/mL), Gibco^®^ Amphotericin B (Fungizone), and 96-Well Black/Clear Bottom Plate and Falcon™ Tissue Culture T75 Flasks were obtained from Thermo Fisher Scientific™ (Thermo Fisher Scientific Inc., Miami, FL, USA). Resazurin sodium salt was purchased from Acros Organics (Thermo Fisher Scientific Inc., NJ, USA). In total, 12 mm of Snapwell^®^ inserts (0.4 μm polyester membrane, 6-well plate) were obtained from Corning, Fisher Scientific, Suwanee, GA, USA). An ENDOHM-24G Chamber Cup (World Precision Instruments, Sarasota, FL, USA) was used to measure transepithelial electrical resistance (TEER).

Primary NHEKs (normal human epidermal keratinocytes) and the KGM™ Gold Keratinocyte Growth Medium BulletKit™ (Culture system containing KBM™ Gold™ Basal Medium and KGM™ Gold™ Single Quots™ supplements) were purchased from Lonza Walkersville Inc., MD, USA. Gibco^®^ Collagen I (Rat Tail), 96-Well Black/Clear Bottom Plate, and Falcon™ Tissue Culture T75 Flasks were obtained from Thermo Fisher Scientific™ (Thermo Fisher Scientific Inc., Miami, FL, USA). Resazurin, sodium salt, was purchased from Acros Organics (Thermo Fisher Scientific Inc., NJ, USA) and Dimethyl Sulfoxide (DMSO) from Millipore-Sigma, St. Louis, MO, USA.

### 4.2. Physicochemical Characterization

#### 4.2.1. Scanning Electron Microscopy (SEM) and Energy-Dispersive X-ray (EDX) Spectroscopy

The SEM and EDX data of raw HI-TOPK-032 were acquired with the Phenom ProX G6 (NanoScience Instruments, ThermoFisher Scientific, Phoenix, AZ, USA) following similar conditions reported previously [17,26]. The sample was mounted on an aluminum stub with a double-sided adhesive carbon tab (Ted Patella, Inc. Redding, CA, USA). The powder sample was coated with a platinum alloy (5 nm) using a Luxor platinum sputter coater (NanoScience Instruments, ThermoFisher Scientific, Phoenix, AZ, USA) under argon plasma (Airgas, Air Liquide, FL, USA). The SEM micrographs were captured using a Secondary Electron Detector (SED) at various magnifications at an accelerating voltage of 10 kV, a working distance of approximately 7 mm, and an intensity set on ‘image’ (Phenom ProX G6 software, NanoScience Instruments, Phoenix, AZ, USA)). The EDX spectrum of Hi-TOPK-032 powder was obtained at an accumulation voltage of 15 kV using a full Secondary Electron Detector at an 8000× *g* magnification.

#### 4.2.2. Differential Scanning Calorimetry (DSC)

The thermal transitions of raw HI-TOPK-032 were determined using a Discovery Differential Scanning Calorimeter 250 with ultra-high purity (UHP) nitrogen gas (Airgas, Air Liquide, Palm Beach, FL, USA) with a flow rate of 50 mL/min and TRIOS v5.6.0.87 software for analysis (DSC250 with T-Zero^®^ Technology (TA Instruments, New Castle, DE, USA). As described in previously published methods [27,28], briefly, 2–5 mg of the sample was packed into hermetic anodized aluminum T-Zero^®^ DSC pan, and aluminum lids were hermetically sealed using a T-Zero^®^ hermetic press (TA Instruments, New Castle, DE, USA). The reference pan was an empty, hermetically sealed T-Zero^®^ aluminum pan. The raw HI-TOPK-032 samples were heated from 0 °C to 300 °C at a scanning rate of 5.00 °C/min. Experiments were performed in quadruplicate (*n* = 4).

#### 4.2.3. Hot-Stage Microscopy (HSM)

Using similar conditions to those described previously, HSM of a raw HI-TOPK-032 powder sample was conducted. A microscopic glass slide containing a powder sample covered with a glass coverslip was placed on a Mettler FP82 hot stage (Columbus, OH, USA) attached to a Mettler FP 80 central processor heating unit and heated from 25.0 °C to 300.0 °C at a heating rate of 5.00 °C/min. Thermo-microscopic changes in the sample were observed under a cross-polarized light microscope (Leica DMLP, Wetzlar, Germany), and images were captured using a digital camera (Nikon Coolpix 8800, Nikon, Tokyo, Japan) under a 10× optical objective and 10× digital zoom.

#### 4.2.4. X-ray Powder Diffraction (XRPD)

The crystallinity of raw HI-TOPK-032 powder was determined by X-ray powder diffraction (XRPD) analysis using similar conditions to those reported previously [17,26,28]. XRPD patterns of raw HI-TOPK-032 powder were recorded using a PANalytical X’pert diffractometer (PANalytical Inc., Westborough, MA, USA) equipped with a programmable incident beam slit and an X’celerator detector at room temperature. The powder sample was loaded onto a zero-background silicon sample holder as a thin layer and scanner over an angular range of 5.0 to 50.0° with a scanning rate of 2.00°/min using Ni-filtered Cu Kα (45 kV, 40 Ma, and λ = 1.5444 Å). All measurements were carried out in triplicate.

#### 4.2.5. Karl Fisher (KF) Coulometric Titration

The residual water content of raw HI-TOPK-032 was analytically determined with Karl Fisher (KF) coulometric titration using similar conditions previously reported by the authors [29]. The measurements were carried out using a TitroLine^®^ 7500 KF trace titrator (SI Analytics, Weilheim, Germany) coupled with a generator electrode TZ 1752 and a micro double platinum electrode KF 1150. Approximately 5 mg of the powder sample was added to the titration cell that contained the Hydranal^TM^ Coulomat AD reagent (Honeywell Fluka^TM^, Seelze, Germany). The residual water content of the sample was then accessed via endpoint titration. All samples were measured in triplicate.

#### 4.2.6. Raman Spectrometry and Confocal Raman Microscopy (CRM)

Utilizing previously reported conditions [30,31,32], Raman acquisitions for molecular fingerprinting were obtained using a 785 nm laser of 30 mW in intensity in the DXR™ Raman system (Thermo Scientific™, Fitchburg, WI, USA) equipped with an Olympus BX41 confocal optical microscope with bright-field illumination (Olympus America, Inc., Chester Valley, PA, USA) and OMNIC™ for Dispersive Raman v9.12.1019 software. Briefly, each spectral point was acquired using 16 sample exposures each, with a detector exposure time of 4 s. A 50 µm confocal hole and 400 lines/mm grating were used. Spectra were baseline-corrected, and smoothing was performed prior to further analysis. All measurements were conducted in triplicate (*n* = 3).

Raman spectral maps were obtained using a 10× objective with 10 µm steps and 3 points each along the x and y axes to acquire 9 individual Raman acquisitions [33,34]. Each map point was acquired using 16 sample exposures with a 4 sec detector exposure time, 50 µm confocal hole, and 400 lines/mm grating. Baseline correction and smoothing were performed on the spectra prior to further analysis. These conditions have been described previously [35,36]. Mapping experiments were conducted in triplicate (*n* = 3).

#### 4.2.7. Attenuated Total Reflectance-Fourier Transform Infrared Spectroscopy (ATR-FTIR) and Fourier Transform Infrared Microscopy

The ATR-FTIR spectroscopy was performed using the Nicolet™ iS50 FTIR spectrometer (Thermo Scientific™, USA) configured with a deuterated triglycine sulfate (DTGS) detector. Each spectrum was acquired with 32 scans at a spectral resolution of 8 cm^−1^ over the wavenumber range of 4000–400 cm^−1^. The same experimental conditions were used to collect a background spectrum. Spectral data were obtained using the OMNIC v9.12.928 software. Baseline corrected and smoothed spectra were used for further analysis. The conditions used here have been reported previously [30,32,37].

FT-IR microscopy for chemical imaging and spectral mapping was performed using a Nicolet™ Continuum™ Infrared Microscope (Thermo Scientific™, USA) equipped with mercury–cadmium–telluride (MCT)/A detector, cooled by liquid nitrogen. Spectral maps were obtained with a 15× objective, step size 10 µm in the x and y direction, and the selected aperture was 100 × 100 μm. Nine individual acquisitions were acquired. Each spectrum was collected with 32 scans, an 8 cm^−1^ spectral resolution, and a wavelength number range of 4000–700 cm^−1^. A background spectrum was collected under the same experimental conditions. Spectral data were acquired using the OMNIC v9.12.928 and the OMNIC Atlµs™ v9.12.990 software. Spectra were subjected to baseline correction and smoothening prior to further analysis.

### 4.3. In Vitro 2D Cell Culture Dose–Response Assay with HaCaT Cells and NHEK Cells

Following previously published growth conditions and methods [17,27,28,32,38], HaCaT cells (immortalized normal human keratinocyte cell line) were grown in collagen Type I-coated (concentration 5–10 μg/cm^2^ in PBS) T-75 flasks using Advanced Dulbecco’s Modified Eagle’s Medium (ADMEM) 1× supplemented with 10% (*v*/*v*) FBS, 0.2% *v*/*v* Fungizone (0.5 μg/mL Amphotericin B, 0.41 μg/mL Sodium Deoxycholate), and 1% *v*/*v* Pen-Strep (100 Unit/mL Penicillin, 100 µg/mL Streptomycin) in a humidified incubator at 37 °C and a 5% CO_2_ atmosphere. At 90% confluence, the HaCaT cells were seeded into a 96-well Black/Optical Bottom Plate at a density of 5000 cells/well in 100 µL of supplemented ADMEM, followed by incubation at 37 °C and 5% CO_2_ for 48 h to allow the attachment of cells to the plate surface. After 48 h, cells were exposed to different HI-TOPK-032 concentrations. The HI-TOPK-032 solution was prepared by dissolving in 100% DMSO to produce an initial HI-TOPK-032 4 mg/mL concentration, which was diluted further with non-supplemented ADMEM. A 100 µL volume of the following drug concentrations was used: 0 µM (control), 0.1 µM, 1 µM, 10 µM, 100 µM, or 1000 µM was added to each well with 48 h exposure time and incubation at 37 °C and 5% CO_2_. At the end of exposure time, the non-supplemented ADMEM with the drug was removed from each well and replaced with 100 µL of non-supplemented ADMEM containing 20 µL of 20 µM resazurin sodium salt dissolved in non-supplemented ADMEM followed by incubation for 4 h at 37 °C and 5% CO_2_. At 4 h, the resorufin fluorescence intensity produced by viable cells was measured at 544 nm (excitation wavelength) and 590 nm (emission wavelength) using the BioTek Synergy H1 Microplate Reader equipped with Gen5 v2.09.2 software (BioTek Instruments Inc., Winooski, VT, USA). The relative % cell viability was calculated using Equation (1).

(1) $$\mathrm{Relative}\;\mathrm{cell}\;\mathrm{viability}\;\left(\%\right)=\frac{\mathrm{Sample}\;\mathrm{fluorescence}\;\mathrm{intensity}\;}{\mathrm{Control}\;\mathrm{fluorescence}\;\mathrm{intensity}}\times 100$$

NHEKs were grown according to the manufacturer’s instructions in a humidified incubator at 37 °C and 5% CO_2_ in a collagen-coated T75 flask. After 90% confluence, 5000 cells/well were seeded in a 96-Well Black/Optical Bottom Plate in 100 µL of supplemented keratinocyte growth medium (KGM) and allowed 48 h for attachment to the plate surface in the humidified incubator at 37 °C and 5% CO_2_. At 48 h, the cells were exposed to increasing concentrations of the raw HI-TOPK-032 drug. A 4 mg/mL raw HI-TOPK-032 stock was prepared in 100% DMSO and diluted further with non-supplemented KGM. The following drug concentrations: 0 µM (control), 0.1 µM, 1 µM, 10 µM, 100 µM, and 1000 µM were added to each well with 48 h exposure time in a humidified incubator at 37 °C and 5% CO_2_ [17] as was conducted previously, to determine the dose-response of NHEKs (*n* = 24). After 48 h, the non-supplemented KGM with the drug was removed from each well and replaced by 100 µL non-supplemented KGM containing 20 µL of 20 µM resazurin sodium salt followed by 4 h humidified incubation at 37 °C and 5% CO_2_. At 4 h, the resorufin fluorescence intensity produced by the viable cells was measured at 544 nm (excitation wavelength) and 590 nm (emission wavelength) using the BioTek Synergy H1 Microplate Reader with Gen5 v2.09.2 software (BioTek Instruments Inc., Winooski, VT, USA). The % relative cell viability was determined using Equation (1).

### 4.4. In Vitro Transepithelial Electrical Resistance (TEER) with Skin Epithelial Cells at Air-Liquid Interface (ALI)

TEER assesses the in vitro membrane barrier tightness and integrity of the cellular membrane by recording the blocked electrical signal through resistance measurements. TEER is an established marker of the tight junction function of cellular layers. HaCaT cells are nontumorigenic, immortalized keratinocytes from normal skin that exhibit normal morphogenesis. Using information from previously published methods [18,27,38], HaCaT cells were grown in supplemented Advanced Dulbecco’s Modified Eagle’s Medium (ADMEM), with 10% FBS, 1% Pen-Strep (100 U/mL penicillin, 100 μg/mL streptomycin), 0.2% Fungizone (0.5 μg/mL amphotericin B, 0.41 μg/mL sodium deoxycholate) and 1% GlutaMAX™ in a humidified incubator at 37 °C and 5% CO_2_. After confluence, cells were seeded at ~400,000 cells per well in 12 mm Snapwell^®^ inserts and ~50,000 cells per well in 6.5 mm Transwell^®^ inserts (0.4 μm polyester membrane, 6-well plate and 24-well plate, Corning, Fisher Scientific, Suwanee, GA, USA) using supplemented ADMEM with appropriate volumes on the apical side and the basal side as per the manufacturer’s guidelines. Supplemented ADMEM was changed every second day from the basal side. After a week, the cells appeared densely packed, forming a monolayer visible via light microscopy, and the transepithelial electrical resistance (TEER) values were measured using ENDOHM-24G and ENDOHM-6G Chamber Cups (World Precision Instruments, Sarasota, FL, USA). Under liquid-covered culture (LCC) conditions, when TEER values reached ~140 Ω·cm^2^ for 12 mm inserts, and ~100 Ω·cm^2^ for 6.5 mm inserts, media from the apical side were removed to facilitate air–liquid interface (ALI) conditions for 72 h. Under ALI conditions, TEER values were monitored when the value was stabilized at ~100 Ω·cm^2^ for 12 mm (~70 Ω·cm^2^ for 6.5 mm), the cells were exposed to 100 μM HI-TOPK-032 (4 mg/mL stock solution in DMSO) diluted using non-supplemented media. TEER values were recorded after 3 h of HI-TOPK-032 exposure with subsequent recordings every 24 h for up to 7 days using ENDOHM-24G and ENDOHM-6G Chamber Cups. TEER values for naïve- (non-treated) and vehicle (DMSO)- treated cells were also recorded simultaneously. TEER was recorded with 0.5 mL media added to each Snapwell^®^ and Transwell^®^ insert and immediately removed to return the cells to ALI conditions. SigmaPlot^®^ v15 (SYSTAT Software, Inc., Palo Alto, CA, USA) was used to plot the TEER values between HI-TOPK-032-treated versus naïve (non-treated) HaCaT cells. All measurements were recorded from four separate cell inserts (*n* = 4 replicates). The plot represents data calculated as the percentage response of the control using Equation (2) [39]:

(2) $$\mathrm{TEER}\;\%\;\mathrm{control}=\frac{\mathrm{Sample}\;\mathrm{TEER}\;\mathrm{Value}}{\mathrm{Control}\;\mathrm{TEER}\;\mathrm{Value}}\times 100\%$$

### 4.5. In Vitro Membrane Permeation of HI-TOPK-032

The in vitro permeation behavior of HI-TOPK-032 from a propylene glycol solution was determined using Franz diffusion cells (PermeGear, Inc., Hellertown, PA, USA), following similar conditions to those reported previously [17,28]. The Strat-M^®^ membrane (Millipore Sigma, Danvers, MA, USA), a synthetic, non-animal-based transdermal diffusion test model membrane that is predictive of diffusion through human skin, was mounted between two O-rings with an orifice of 0.64 cm^2^ and sandwiched between the donor and receptor chambers with a clamp. The receptor chamber was filled with 5 mL of freshly prepared phosphate-buffered saline (PBS, pH 7.4) containing Tween^®^ 80 (5% *w*/*v*). Tween 80 was used as a solubilizer in the receptor medium to maintain sink conditions. The diffusion cells were maintained at 35 °C ± 0.05 °C using a precision reciprocal shaking bath model 25 (Thermo Fisher Scientific, Fair Lawn, NJ, USA) with 30 oscillations per minute. The HI-TOPK-032 solution was prepared by dissolving 1 mg of the drug in 0.4 mL of DMSO with the aid of sonication for 10 min followed by the addition of 0.6 mL propylene glycol. An aliquot of 200 µL of the drug solution was added to the donor compartment, and 200 μL samples were collected from the receptor chamber at 0.5, 1.0, 2.0, 3.0, 4.0, 5.0, and 6.0 h time intervals and replaced with an equal volume of the fresh medium. The samples were then analyzed using HPLC after appropriate dilution using methanol. The permeation experiments were conducted in triplicate. The cumulative amounts of drug permeated (µg/cm^2^) were plotted as a function of time, and the flux at a steady state (J) was determined as the slope of linear regression analysis for the linear portion of the permeation curve [17,40].

### 4.6. High-Performance Liquid Chromatography (HPLC) Method Development, Optimization, and Validation

All HPLC runs were performed using a reverse-phase high-performance liquid chromatography (HPLC) LC 2050C 3D system (Shimadzu, Kyoto, Japan) equipped with the Luna^®^ C_18_ silica column, 100 Å, 250 × 4.6 mm (Phenomenex, Torrance, CA, USA) maintained at 30 °C. This system was operated, and results were acquired and processed by LabSolutions software (Version 5.110) to control the instrument parameters.

Chromatographic analysis of HI-TOPK-032 was performed in the isocratic mode. The mobile phase consisted of 25:75 (% *v*/*v*) water and a mixture of methanol and acetonitrile (50:50 *v*/*v*), which was pumped at a flow rate of 1 mL/min. The sample injection volume was 10 µL, and the detection wavelength was 205 nm. The total run time was 7.5 min, and the total area of the peak was used for drug quantification. A mixture of methanol and acetonitrile (50:50 *v*/*v*) was used as a diluent.

#### 4.6.1. Preparation of Calibration Standard and Quality Control (QC) Samples

A stock solution of HI-TOPK-032 was prepared by dissolving 1 mg of the accurately weighed drug in 0.4 mL of DMSO with the aid of sonication for 10 min, followed by dilution to 1 mL using a mixture of methanol/ acetonitrile (50:50 *v*/*v*) as a diluent. Standard solutions of HI-TOPK-032 with drug concentrations in the range of 5–80 µg/mL were prepared through the dilution of the stock solution with a diluent. An aliquot of 100 µL of the standard solution was transferred into microcentrifuge tubes (1.6 mL) and diluted to a final volume of 1 mL with the diluent to prepare calibration standards with drug concentrations of 0.5, 1, 2, 4, 6, and 8 µg/mL. Three QC samples were at concentrations of 0.5, 5, and 8 µg/mL representing the low, medium, and high concentrations, respectively.

#### 4.6.2. Assay Validation

Assay validation was carried out according to the International Conference on Harmonization (ICH) guidelines [41].

#### 4.6.3. System Suitability, Linearity and Sensitivity

The system’s suitability was evaluated using six replicate injections of standard solution at 5 µg/mL of HI-TOPK-032. The percentage coefficient of variation (%CV) for the peak retention time, peak area, peak height, the number of theoretical plates, and tailing factor was determined with an acceptance criterion of ±2%.

The linearity was determined by the construction of calibration curves using the calibration standards in triplicate. Linearity was evaluated by linear regression analysis and calculated by the least square regression method.

The sensitivity of the developed analytical method was determined by estimating the limit of detection (LOD) and limit of quantification (LOQ) from the signal-to-noise ratio. The LOD indicates the lowest concentration level, resulting in a peak area of three times the baseline noise. The LOQ indicates the lowest concentration level that provides a peak area with a signal-to-noise ratio higher than 10, with precision (% CV) and accuracy (% bias) within ± 10%. LOD and LOQ were calculated using Equations (3) and (4):

(3) $$LOD=\frac{3.3\sigma }{S}$$

(4) $$LOQ=\frac{10\sigma }{S}$$

where σ is the standard deviation of the peak response at the lowest concentration of a regression line and S is the slope of the calibration curve.

#### 4.6.4. Accuracy, Precision and Recovery

Intra-day and inter-day precision (as relative standard deviation, RSD), accuracy (as concentration bias (%)), and recovery (%) were determined using an assay of 6 replicates of QC samples on 3 different days. The RSDs for intra-day precision were calculated using the mean values of 6 replicates at each concentration for a single day, and inter-day RSDs were calculated from the mean value of 18 determinations on 3 different days. The bias (%) was calculated as 100 × (nominal concentration − measured concentration)/nominal concentration). Recovery was determined through a comparison of the measured concentration to the nominal concentration (0.5, 5, and 10 µg/mL of QC samples).

#### 4.6.5. Robustness, Carry-Over and Stability

The robustness of the developed analytical method was evaluated by injecting QC samples with a deliberate variation of ±5% units of the mobile phase flow rate (1 mL/min) and the column temperature (30 °C). The effect of variation on the peak areas, retention times, found drug concentrations, the number of theoretical plates, and tailing factor were evaluated.

Carry-over was determined through the injection of a diluent directly following a high QC sample run.

The short-term stability of the drug solution was tested by an analysis of triplicate low and high QC samples during storage for 24 h and 48 h under the following conditions: on the bench at an ambient temperature (~20 °C); in autosampler vials at 15 °C; in a refrigerator at 4 °C; in a freezer at −20 °C.

### 4.7. Statistical Analysis

Data (mean ± standard deviation (SD)) were subjected to one-way analysis of variance (ANOVA) and Student–Newman–Keuls post hoc testing using Sigma Plot^®^ version 15.0 (Systat Software Inc., San Jose, CA, USA). A probability level of 5% (*p* ≤ 0.05) was considered statistically significant.

## 5. Conclusions

In conclusion, this systematic and comprehensive study reports a complete physicochemical characterization of a new TOPK inhibitor, HI-TOPK-032, for the first time. The physicochemical evaluation results confirmed the amorphous nature of the drug and the homogeneity of the sample with all characteristic chemical peaks. The in vitro cell viability assay results confirmed 100% cell viability for up to 10 µM of HI-TOPK-032, demonstrating biocompatibility as a function of drug dose. An isocratic reversed-phase HPLC assay with a photodiode array detector for the quantification of HI-TOPK-032 was developed and validated. This method is simple, accurate, and precise without the use of an internal standard. This method was used to evaluate the drug permeation behavior of HI-TOPK-032 using the Strat-M^®^ synthetic biomimetic membrane.

## Figures and Tables

**Table 1:** DSC thermal analysis data (*n* = 4, mean ± standard deviation).

Raw HI-TOPK-032				
Endotherm 1		Endotherm 2		Exotherm
T_peak_ (°C)	Enthalpy (J/g)	T_peak_ (°C)	Enthalpy (J/g)	T_peak_ (°C)
145.26 ± 3.4	0.3 ± 0.12	171.19 ± 3.41	1.26 ± 0.08	216.54 ± 0.95

**Table 2:** Spectral peaks from Raman spectrometry and FTIR spectrometry of HI-TOPK-032 (*n* = 3).

Raman Spectrometry Peaks (cm^−1^)	FTIR Spectrometry Peak (cm^−1^)	
785.31	446.63	1257.69
1638.61	487.36	1283.93
1677.49	522.66	1354.18
2206.83	604.27	1412.92
	629.77	1430.98
	652.86	1459.59
	720.42	1489.19
	758.16	1521.73
	802.25	1564.88
	859.32	1643.51
	1029.93	1673.75
	1062.59	2203.82
	1087.76	3092.39
	1126.6	3296.18
	1206.07	

**Table 3:** (A) System suitability parameters of the validated analytical method for HI-TOPK-032 (5 µg/mL). (B) Linearity and sensitivity results of HI-TOPK-032.

(A)					
	HI-TOPK-032 (5 µg/mL)				
	Retention Time (min)	Peak Area	Peak Height (mAU)	Number of Theoretical Plates (USP)	Tailing Factor (10%)
Mean	4.3733	6,892,319.33	927,983.83	7630.67	1.3362
S.D.	0.0005	9727.43	767.37	1.25	0.0004
%CV	0.0108	0.1411	0.0827	0.0163	0.0279
**(B)**					
		**Mean ± S.D. (*n* = 6)**			
Slope		1,445,602 ± 7756			
Intercept		114,432 ± 12,582			
Correlation coefficient (*r*^2^)		0.9993 ± 0.0002			
LOD (µg/mL)		0.010			
LOQ (µg/mL)		0.030			

CV, coefficient of variation, LOD, limit of detection, LOQ, limit of quantification.

**Table 4:** Intra- and inter-day accuracy and precision values at different concentration levels for the validated analytical method.

	Conc Level	Found Conc. (µg/mL)	% Recovery	% RSD	% Bias
Day 1	LQC (0.5 µg/mL)	0.511 ± 0.008	102.11 ± 1.61	1.58	−2.11
	MQC (5.0 µg/mL)	4.886 ± 0.002	97.72 ± 0.04	0.05	2.28
	HQC (8.0 µg/mL)	8.245 ± 0.027	103.06 ± 0.34	0.33	−3.06
Day 2	LQC (0.5 µg/mL)	0.482 ± 0.014	96.32 ± 2.79	2.90	3.68
	MQC (5.0 µg/mL)	5.171 ± 0.064	103.41 ± 1.28	1.24	−3.41
	HQC (8.0 µg/mL)	8.256 ± 0.097	103.20 ± 1.21	1.17	−3.20
Day 3	LQC (0.5 µg/mL)	0.476 ± 0.009	95.13 ± 1.79	1.88	4.88
	MQC (5.0 µg/mL)	4.949 ± 0.022	98.98 ± 0.44	0.45	1.02
	HQC (8.0 µg/mL)	7.676 ± 0.031	95.95 ± 0.38	0.40	4.05

**Table 5:** Robustness of the validated analytical method.

Parameter	Conc. Level	Retention Time (min)		Peak Area		Number of Theoretical Plates (USP)		Tailing Factor (10%)		Found Conc. (µg/mL)		
		Mean ± S.D.	%RSD	Mean ± S.D.	%RSD	Mean ± S.D.	%RSD	Mean ± S.D.	%RSD	Mean ± S.D.	%RSD	%Bias
Change in mobile phase flow rate												
0.95 mL/min	LQC (0.5 µg/mL)	4.596 ± 0.002	0.052	831,928 ± 5673	0.682	8100.333 ± 48.016	0.593	1.313 ± 0.003	0.257	0.496 ± 0.004	0.791	−0.734
	MQC (5.0 µg/mL)	4.592 ± 0.002	0.053	8,067,694 ± 137,430	1.703	7792.778 ± 29.604	0.380	1.336 ± 0.002	0.1158	5.502 ± 0.095	1.728	10.034
	HQC (8.0 µg/mL)	4.592 ± 0.002	0.043	12,078,181 ± 96,746	0.801	7418.111 ± 46.620	0.628	1.344 ± 0.001	0.089	8.276 ± 0.067	0.809	3.450
1.00 mL/min	LQC (0.5 µg/mL)	4.373 ± 0.003	0.065	788,198 ± 5017	0.637	7819.111 ± 50.132	0.641	1.319 ± 0.004	0.334	0.466 ± 0.003	0.745	−6.784
	MQC (5.0 µg/mL)	4.369 ± 0.002	0.054	7,658,981 ± 131,529	1.717	7493.667 ± 67.180	0.896	1.342 ± 0.003	0.245	5.219 ± 0.091	1.743	4.379
	HQC (8.0 µg/mL)	4.369 ± 0.002	0.040	11,465,342 ± 108,979	0.951	7126.889 ± 53.813	0.755	1.353 ± 0.003	0.236	7.852 ± 0.075	0.960	−1.850
1.05 mL/min	LQC (0.5 µg/mL)	4.162 ± 0.003	0.063	756,240 ± 7580	1.002	7444.778 ± 41.593	0.559	1.327 ± 0.003	0.244	0.444 ± 0.005	1.181	−11.205
	MQC (5.0 µg/mL)	4.159 ± 0.003	0.061	7,307,762 ± 125,068	1.711	7227.000 ± 41.331	0.572	1.345 ± 0.003	0.216	4.976 ± 0.087	1.739	−0.480
	HQC (8.0 µg/mL)	4.159 ± 0.002	0.051	10,947,235 ± 92,631	0.846	6857.222 ± 56.790	0.828	1.352 ± 0.002	0.139	7.494 ± 0.064	0.855	−6.330
Change in oven temperature												
29 °C	LQC (0.5 µg/mL)	4.383 ± 0.003	0.070	792,882 ± 6295	0.794	7742.778 ± 47.316	0.611	1.319 ± 0.005	0.349	0.469 ± 0.004	0.928	−6.136
	MQC (5.0 µg/mL)	4.381 ± 0.002	0.054	7,674,089 ± 127,472	1.661	7469.333 ± 47.617	0.637	1.343 ± 0.003	0.201	5.229 ± 0.088	10.686	4.588
	HQC (8.0 µg/mL)	4.379 ± 0.002	0.043	11,492,951 ± 100,200	0.872	7102.222 ± 49.207	0.693	1.353 ± 0.002	0.181	7.871 ± 0.069	0.881	1.611
30 °C	LQC (0.5 µg/mL)	4.373 ± 0.003	0.065	788,198 ± 5017	0.637	7819.111 ± 50.132	0.641	1.319 ± 0.004	0.334	0.466 ± 0.003	0.745	−6.784
	MQC (5.0 µg/mL)	4.369 ± 0.002	0.054	7,658,981 ± 131,529	1.717	7493.667 ± 67.180	0.896	1.342 ± 0.003	0.245	5.219 ± 0.091	1.743	4.379
	HQC (8.0 µg/mL)	4.369 ± 0.002	0.040	11,465,342 ± 108,979	0.951	7126.889 ± 53.813	0.755	1.353 ± 0.003	0.236	7.852 ± 0.075	0.960	−1.850
31 °C	LQC (0.5 µg/mL)	4.360 ± 0.003	0.060	791,434 ± 8044	1.016	7789.333 ± 50.607	0.650	1.319 ± 0.004	0.331	0.468 ± 0.006	0.331	−6.336
	MQC (5.0 µg/mL)	4.357 ± 0.003	0.061	7,669,947 ± 126,954	1.655	7472.556 ± 65.934	0.882	1.342 ± 0.002	0.153	5.227 ± 0.088	1.680	4.531
	HQC (8.0 µg/mL)	4.356 ± 0.002	0.037	11,485,631 ± 95,252	0.829	7129.556 ± 44.199	0.620	1.351 ± 0.004	0.302	7.866 ± 0.066	0.838	−1.674

**Table 6:** Short-term stability of HI-TOPK-032 in quality control (QC) samples under different storage conditions (data are means, *n* = 3).

Condition		Conc Level	Found Conc. (µg/mL)	% Accuracy	% RSD	% Bias
Bench top	24 h	LQC (0.5 µg/mL)	0.496 ± 0.002	99.226 ± 0.438	0.442	0.774
		HQC (8.0 µg/mL)	8.286 ± 0.019	103.579 ± 0.243	0.235	−3.579
	48 h	LQC (0.5 µg/mL)	0.520 ± 0.001	103.903 ± 0.180	0.173	−3.903
		HQC (8.0 µg/mL)	7.812 ± 0.018	97.655 ± 0.227	0.233	2.345
Autosampler (15 °C)	24 h	LQC (0.5 µg/mL)	0.499 ± 0.002	99.705 ± 0.344	0.910	0.295
		HQC (8.0 µg/mL)	8.272 ± 0.075	103.397 ± 0.941	0.910	−3.397
	48 h	LQC (0.5 µg/mL)	0.517 ± 0.005	103.427 ± 0.960	0.928	−3.427
		HQC (8.0 µg/mL)	7.962 ± 0.023	99.521 ± 0.292	0.293	0.479
Refrigeration at 4 °C	24 h	LQC (0.5 µg/mL)	0.476 ± 0.004	95.190 ± 0.783	0.822	4.810
		HQC (8.0 µg/mL)	7.938 ± 0.218	99.228 ± 2.722	2.743	0.772
	48 h	LQC (0.5 µg/mL)	0.510 ± 0.004	101.980 ± 0.824	0.808	−1.980
		HQC (8.0 µg/mL)	7.745 ± 0.057	96.817 ± 0.708	0.732	3.183
Freezer at −20 °C	24 h	LQC (0.5 µg/mL)	0.491 ± 0.001	98.182 ± 0.122	0.124	1.818
		HQC (8.0 µg/mL)	8.151 ± 0.044	101.887 ± 0.548	0.538	−1.887
	48 h	LQC (0.5 µg/mL)	0.512 ± 0.009	102.401 ± 1.797	1.755	−2.401
		HQC (8.0 µg/mL)	7.883 ± 0.044	98.533 ± 0.547	0.555	1.467

## Data Availability

The data presented in this study are available on request from the corresponding author.
